# SASH1 mediates sensitivity of breast cancer cells to chloropyramine and is associated with prognosis in breast cancer

**DOI:** 10.18632/oncotarget.12020

**Published:** 2016-09-14

**Authors:** Joshua T. Burgess, Emma Bolderson, Jodi M. Saunus, Shu-Dong Zhang, Lynne E. Reid, Anne Marie McNicol, Sunil R. Lakhani, Katharine Cuff, Kerry Richard, Derek J. Richard, Kenneth J. O'Byrne

**Affiliations:** ^1^ Cancer and Ageing Research Program, Institute of Health and Biomedical Innovation at the Translational Research Institute (TRI), Queensland University of Technology, Brisbane, Australia; ^2^ Princess Alexandra Hospital, Woolloongabba, Brisbane, Queensland, Australia; ^3^ The University of Queensland (UQ), UQ Centre for Clinical Research, Herston, Queensland, Australia; ^4^ QIMR Berghofer Medical Research Institute, Herston, Queensland, Australia; ^5^ Northern Ireland Centre for Stratified Medicine, University of Ulster, Altnagelvin Hospital Campus, Londonderry, UK; ^6^ Center for Cancer Research and Cell Biology, Queen's University Belfast, United Kingdom; ^7^ Pathology Queensland, Royal Brisbane Women's Hospital, Herston, Queensland, Australia; ^8^ UQ School of Medicine, Herston, Queensland, Australia; ^9^ Translational Cell Imaging Queensland, Translational Research Institute, Queensland, Australia; ^10^ Conjoint Endocrine Laboratory, Pathology Queensland, Queensland Health, Herston, Australia

**Keywords:** SASH1, biomarker, breast cancer, chloropyramine

## Abstract

Expression of the SASH1 protein is reduced in a range of human cancers and has been implicated in apoptotic cancer cell death. This study investigated whether increasing SASH1 expression could be a useful therapeutic strategy in breast cancer. Ectopic SASH1 expression increased apoptosis in 7/8 breast cancer cell lines. Subsequent *in silico* connectivity screening demonstrated that the clinically approved antihistamine drug, chloropyramine, increased *SASH1* mRNA levels. Chloropyramine has previously been shown to have anti-tumour activity in breast cancer in part through modulation of FAK signalling, a pathway also regulated by SASH1. This study demonstrated that chloropyramine increased SASH1 protein levels in breast cancer cells. Consistent with this the agent reduced cell confluency in 7/8 cell lines treated irrespective of their ER status but not apoptosis incompetent MCF7 cells. In contrast *SASH1* siRNA-transfected breast cancer cells exhibited reduced chloropyramine sensitivity. The prognostic significance of *SASH1* expression was also investigated in two breast cancer cohorts. Expression was associated with favourable outcome in ER-positive cases, but only those of low histological grade/proliferative status. Conversely, we found a very strong inverse association in HER2+ disease irrespective of ER status, and in triple-negative, basal-like cases. Overall, the data suggest that SASH1 is prognostic in breast cancer and could have subtype-dependent effects on breast cancer progression. Pharmacologic induction of SASH1 by chloropyramine treatment of breast cancer warrants further preclinical and clinical investigation.

## INTRODUCTION

Breast cancer is the second most common cancer worldwide, comprising 25% of all female cancers. Current therapeutic strategies are based primarily on primary tumour histopathology. Expression of hormone (oestrogen (ER) and progesterone) and human epidermal growth factor 2 (HER2) receptors is routinely assessed in diagnostic practice, as these proteins are strongly prognostic and predict responsiveness to hormone- and HER2-targeted therapies, respectively. Owing to improved management strategies, the survival rate has increased in the past few decades, however breast cancer is still the second highest cause of cancer-associated death in women [1]. For patients who experience distant relapse, secondary disease becomes increasingly harder to control with each line of therapy, with fewer treatment options due to efficient clonal adaptation by the tumour in response to new selection pressures. A key research priority is to identify new tumour cell sensitivities and drug targets, along with companion diagnostic markers to enable further personalisation of therapy, achieving maximal efficacy while minimising over-treatment. Furthermore, deeper molecular and genetic understanding of breast tumourigenesis is intensifying the focus on drug repositioning as a means of capitalising on existing resources, and fast-tracking clinical development of ‘new’ treatments.

SAM and SH3 domain containing 1 (*SASH1*) was initially identified as a putative tumour suppressor gene, based on detection of significantly lower mRNA levels in lung, thyroid and colorectal cancers compared to adjacent normal tissue [2, 3]. Low expression correlates with poor prognosis in colon cancer [4] and glioma [5]. A tumour suppressive role would also be consistent with studies demonstrating that SASH1 depletion increases lung cancer cell line viability, proliferation and migration [3, 6–8], and several recent studies demonstrated that it opposes mesenchymal differentiation and invasive cell behaviour in hepatocarcinoma, thyroid and ovarian cancer cell lines [9–11]. The precise molecular functions of SASH1 in normal tissues and cancer are still being investigated, though it is known to localise to the nucleus, and its SAM and SH3 domains imply signalling, adaptor and/or molecular scaffold functions [12, 13]. Indeed, it can regulate signalling through focal adhesion kinase (FAK) and AKT/PI3K [9, 11] and overexpression promotes apoptosis [3, 6]. SASH1 mRNA and protein levels are also reduced in breast cancer compared to matching normal mammary epithelia [2, 14], with one study suggesting that promoter hypermethylation correlates with repression [14], but the expression and prognostic significance of SASH1 have not yet been investigated in breast tumour cohorts with appreciable clinical annotation or statistical power.

In this study we used *in silico* connectivity mapping and *in vitro* modelling to identify drugs that could be repositioned to augment SASH1 expression in cancer. We found that the antihistamine chloropyramine induced SASH1-dependent cell death in a panel of breast cancer cell lines. In order to identify breast cancer subgroups that could potentially benefit from such a strategy, we analysed the relationships between SASH1 expression, genomic status and clinicopathologic parameters in three large breast tumour cohorts, identifying significant but subtype-dependent relationships between SASH1 expression, relapse and survival. These data suggest that further studies investigating repositioning of chloropyramine are warranted.

## RESULTS

### Increasing SASH1 expression is sufficient to induce breast cancer cell line death

**Figure 1:**
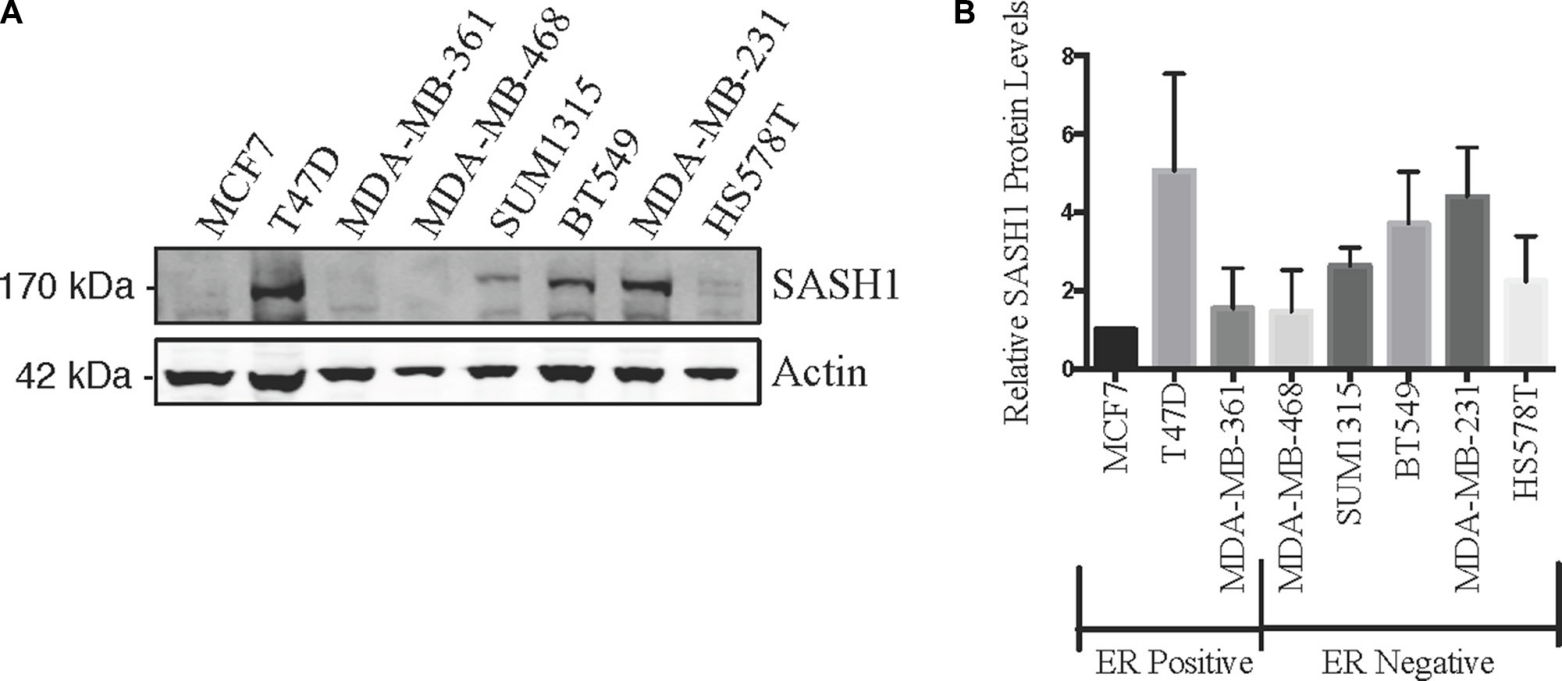
SASH1 protein expression in breast cancer cell lines Breast cancer cell lines were analysed for expression of SASH1 by immunoblotting. Representative immunoblot is shown in (**A**), and (**B**) shows densitometric quantification of SASH1 expression relative to β-actin. Data shown are means +/− standard deviation from three independent experiments, arbitrarily normalised to MCF7.

**Figure 2:**
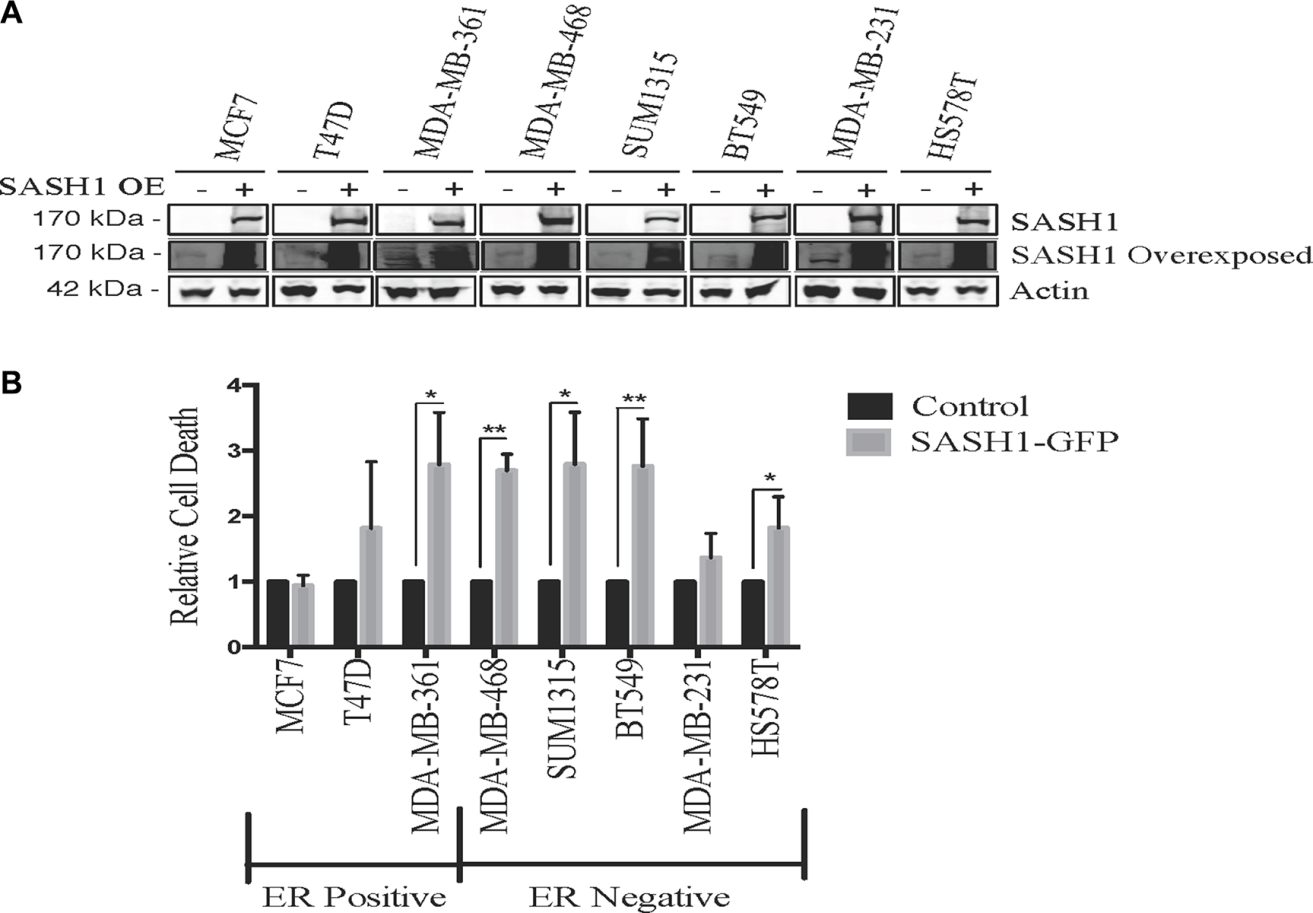
Ectopic SASH1 expression increases cell death (**A**) Confirmation of SASH1 overexpression by immunoblotting. Breast cancer cell lines were transfected with expression constructs encoding a pCMV6-SASH1-GFP fusion protein or pCMV6-GFP alone, then harvested after 48 h for lysate preparation and SASH1/β-actin immunoblotting. Over-expression (OE) (**B**) SASH1 overexpression increases breast cancer cell line death. Cell lines were transfected as above, then stained with Hoechst 33342 and propidium iodide (PI) after 48 h and imaged and quantified using Incell 2200. Data shown are the mean relative proportions of GFP-positive, PI-positive (dead and late apoptotic) cells +/− standard deviation from three independent experiments. Differences between SASH1-GFP and GFP control cultures were assessed using two-tailed *t*-tests. *p < 0.05, **p < 0.005.

We initially quantified SASH1 protein expression in eight breast cancer cell lines by immunoblot analysis. This revealed variable expression, with three high expressing cell lines, T47-D, BT-549 and MDA-MB-231, two moderately expressing lines, Hs578T and SUM-315 and three low expressing lines MCF7, MDA-MB-361 and MDA-MB-468 (Figure 1A–1B). SASH1 has been described as a tumour suppressor, with overexpression resulting in an increase in cell death in lung cancer, melanoma, osteosarcoma and glioma cell lines [3, 6–8]. To investigate this a SASH1-GFP fusion protein was transiently over-expressed in breast cancer cell lines. Overexpression resulted in cell death in 7 of the 8 lines tested (statistically significant in 5 lines), with only the Caspase 3-deficient MCF7 cells showing no response (Figure 2).

### Chloropyramine treatment is sufficient to induce SASH1 expression and apoptosis in breast cancer cell lines

Hypothesising that increasing SASH1 levels may be a novel approach to cancer therapy, we utilised a connectivity screen using the cmap database (Broad Institute [15]) to identify drugs that lead to induction of *SASH1*. This identified a direct correlation between chloropyramine treatment and *SASH1* mRNA expression (*p* = 0.000005, z-score 2.431). Chloropyramine is a first generation reversible H1-receptor antagonist that is approved in several European countries for management of allergic conditions such as conjunctivitis and bronchial asthma.

**Figure 3:**
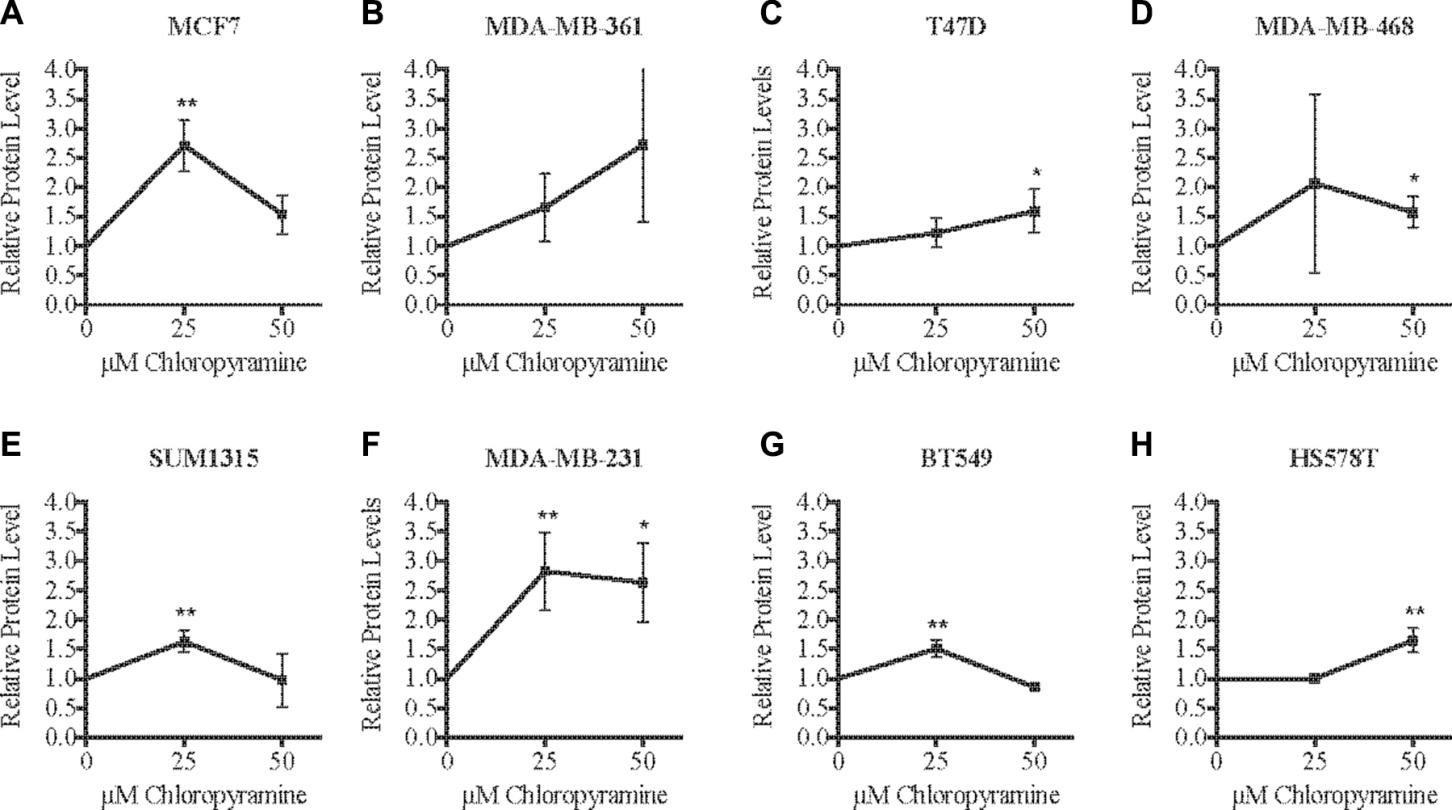
Chloropyramine increases SASH1 expression in breast cancer cell lines (**A**–**H**) Cells were treated with 25 or 50 μM chloropyramine for 24 h, then lysates were prepared and SASH1 protein expression was analysed using immunoblotting. Immunoblot band intensities were quantified relative to β-actin in three independent experiments. The reproducibility and significance of changes in SASH1 expression with treatment were assessed using two-tailed *t*-tests. *p < 0.05, **p < 0.005.

**Figure 4:**
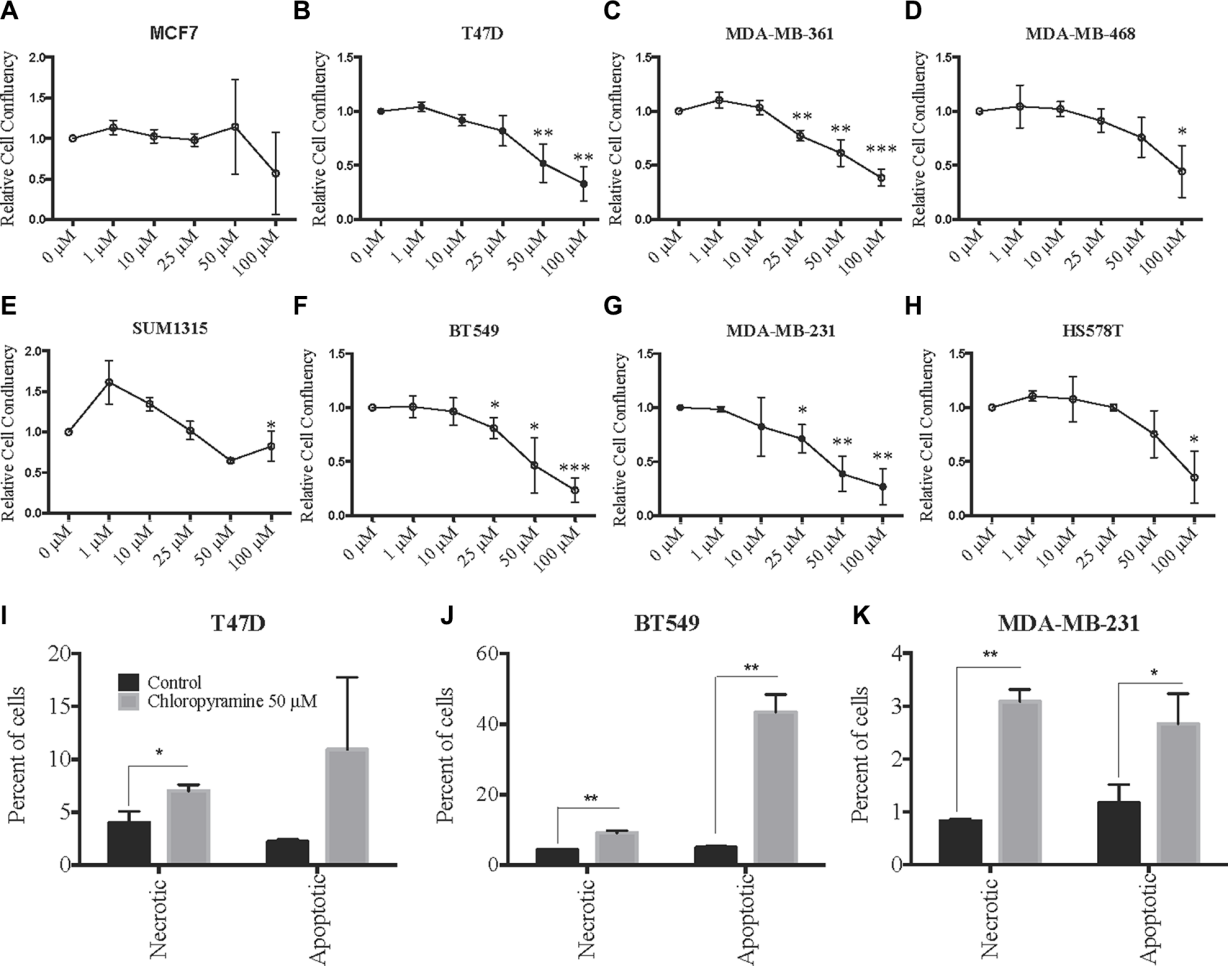
Chloropyramine induces dose-dependent reduction of breast cancer cell line growth that involves apoptosis (**A**–**H**) Changes in adherent breast cancer cell line confluence following chloropyramine treatment. Cells were treated with chloropyramine for 96 h and imaged using light microscopy IncuCyte ZOOM system and digitally analysed to assess confluence relative to an untreated control culture. (**I**–**K**) Chloropyramine induces apoptosis in breast cancer cell lines. Cells were stained with propidium iodide and an Annexin V-FITC antibody conjugate 48 h post-chloropyramine treatment, and analysed by flow cytometry. All data shown are means +/− the standard deviation from three independent experiments. Statistical analysis was performed using two-tailed *t*-tests; *p < 0.05, **p < 0.005, ***p < 0.0005.

**Figure 5:**
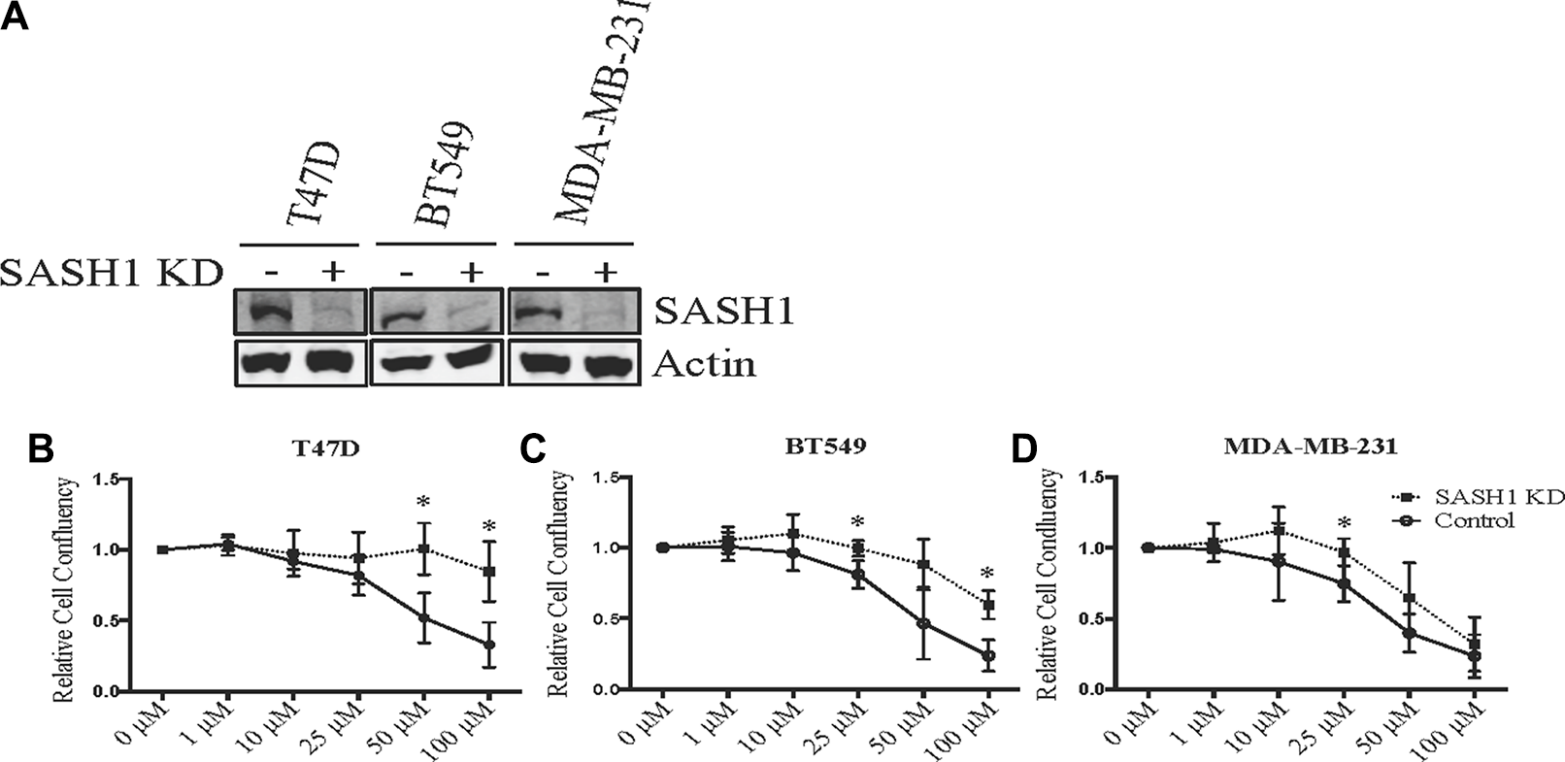
*SASH1* depletion partially rescues chloropyramine-induced apoptosis in breast cancer cell lines (**A**) Cells were transfected with negative control or *SASH1* esiRNAs. After 72 h, cell lysates were prepared and SASH1 expression was analysed relative to β-actin by immunoblotting. Knockdown (KD). (**B**–**D**) Cells were transfected as above, and chloropyramine was added 24 h post-transfection. Cultures were imaged by light microscopy IncuCyte ZOOM system and digitally analysed to assess confluence relative to the untreated control at 96 h post-treatment. Data shown are means +/− the standard deviation from three independent experiments. *t*-tests were used to compare cell confluence with and without *SASH1* depletion at each of the chloropyramine doses; *p < 0.05.

After validating the chloropyramine-mediated induction of SASH1 in breast cancer cell lines at the protein level (Figure 3), we investigated whether this treatment could mimic the effect of SASH1 over-expression on cell growth and survival. Treatment with chloropyramine inhibited cell growth in 7 of the 8 lines treated (Figure 4A–4H). To investigate whether this was due to induction of apoptosis, we analysed post-treatment levels of Annexin V in the three most sensitive cell lines, T47-D, MDA-MB-231 and BT-549. All three lines exhibited an increase in Annexin V (Figure 4I–4K), indicating induction of apoptosis. To determine whether the chloropyramine-induced cell death was SASH1-dependent, we transfected T47-D, MDA-MB-231 and BT-549 cells with SASH1-targeted siRNA prior to treatment (Figure 5A). This experiment demonstrated that SASH1 depletion partially rescued the cell death response in all three lines (Figure 5B–5D), suggesting chloropyramine-induced cell death is at least in part dependent upon SASH1 function.

### SASH1 mRNA and protein levels stratify outcome in breast cancer

**Figure 6:**
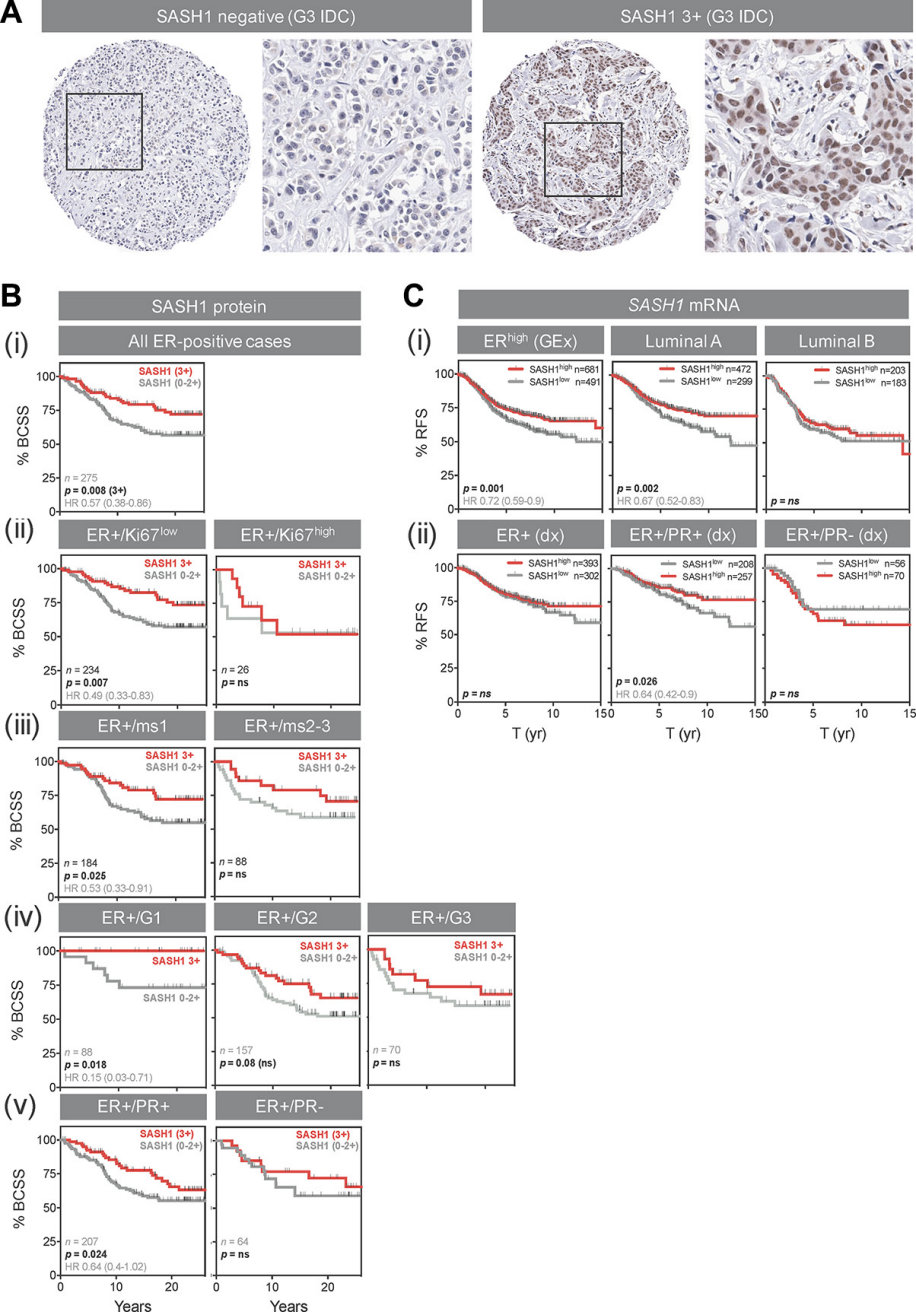
High SASH1 expression is an independent marker of favourable prognosis in ER-positive breast cancer, particularly for low grade and PR co-expressing tumours (**A**) Representative SASH1 IHC images of breast cancer tissue microarray cores. Two grade-3 (G3) invasive ductal carcinomas (IDC) with negative and strongly positive nuclear SASH1 expression are shown at low and high magnification. (**B**) Kaplan-Meier analysis of the relationships between *SASH1* protein expression and breast cancer-specific survival (BCSS) in ER+ breast cancer (defined using current clinical diagnostic (Dx) criteria of positivity in at least 1% of tumour cell nuclei). Across all 275 cases, 3+ SASH1 staining was associated with better long-term survival (i), but this result was driven by tumours characterised by relatively low levels of proliferation (ii, low Ki67 expression; iii, low mitotic score (ms); iv, low-grade) or those with strong co-expression of PR (v). (**C**) Stratification of relapse-free survival according to high or low *SASH1* mRNA expression (KM Plotter database). (i) ER+, luminal A and luminal B cohorts defined by gene expression data. (ii) ER+, ER+/PR+ and ER+/PR- cohorts defined by clinical diagnostic criteria. Log-rank *p* values and hazard ratios (HR; 95% confidence intervals in parentheses) are indicated.

Reasoning that negative or low SASH1 expression may be a predictive biomarker for chloropyramine, we assessed its prognostic significance in the Queensland follow-up cohort (*n* = 449 invasive breast tumours sampled in duplicate on tissue microarrays (TMAs), with clinical annotation including long-term survival outcome [16, 17]). After immunohistochemical (IHC) staining of the TMAs with a well-characterised SASH1 antibody, we observed reasonably homogeneous nuclear staining of breast tumour cells, and scored this as negative, weakly, moderately or strongly positive (Figure 6A; 0, 1, 2 or 3+, respectively).

Next, we analysed the relationships between SASH1 expression and breast cancer-specific survival by Kaplan-Meier analysis, separating the cohort into clinically-relevant subgroups. We found a strong association between 3+ nuclear SASH1 and favourable outcome in ER+ cases (Figure 6B (i); *p* = 0.008; HR 1.7 [1.16–2.67]). The proportions of SASH1-high and –low cases were similar in ER+ subgroups with high or low proliferative activity (*p* = ns; data not shown), but stratification of outcome was restricted to ER+ tumours with low proliferative activity (low levels of Ki67, low mitotic score or low histological grade; Figures 6B (i–iv)) and those co-expressing progesterone receptor (Figure 6B (v)). Chi square analysis showed that SASH1 was moderately associated with ER status, but none of the other clinicopathologic parameters available (Table 1), suggesting independent prognostic value. Consistent with this, multivariate survival analysis of ER+ cases using a stepwise Cox regression model including HER2 status, Ki67 status, tumour size and histological grade revealed that SASH1 expression was independently associated with breast cancer specific survival (BCSS) (Table 2; *HR* = 0.45; 95% confidence interval 0.27–0.77; *p* = 0.0037).

**Table 1:** Relationships between SASH1 protein expression and clinicopathologic indicators in breast cancer using the Queensland follow-up (QFU) cohort

		*n*				% cases			*p* value
	SASH1 staining:	Total	Negative	weak-mod	strong	negative	weak-mod	strong	
Histological type	IDC	228	56	79	93	24.6	34.6	40.8	*ns*
	Lobular/variants	44	9	17	18	20.5	38.6	40.9	
	Mixed ducto-lob	31	8	10	13	25.8	32.3	41.9	
	Mixed	34	4	13	17	11.8	38.2	50.0	
	Metaplastic	15	4	6	5	26.7	40.0	33.3	
	Special types	27	6	13	8	22.2	48.1	29.6	
	*n*	*379*							
Grade	1	51	8	18	25	15.7	35.3	49.0	*ns*
	2	182	43	72	67	23.6	39.6	36.8	
	3	146	36	48	62	24.7	32.9	42.5	
	*n*	*379*							
Age	> 50 yr	250	54	94	102	21.6	37.6	40.8	*ns*
	≤ 50 yr	119	27	40	52	22.7	33.6	43.7	
	*n*	*369*							
Lymph node status	Negative	113	28	38	47	24.8	33.6	41.6	*ns*
	Positive	98	23	46	29	23.5	46.9	29.6	
	*n*	211							
Tumour size	< 2 cm	157	43	49	65	27.4	31.2	41.4	*ns*
	2–5 cm	141	25	57	59	17.7	40.4	41.8	
	> 5 cm	29	5	11	13	17.2	37.9	44.8	
	*n*	*327*							
Lymphovascular invasion	Absent	283	66	107	110	23.3	37.8	38.9	*ns*
	Present	95	20	31	44	21.1	32.6	46.3	
	*n*	*378*							
Lymphocytic infiltrate	Absent	136	34	55	47	25.0	40.4	34.6	*ns*
	Mild	162	36	53	73	22.2	32.7	45.1	
	Moderate-severe	80	17	30	33	21.3	37.5	41.3	
	*n*	*378*							
Central scarring/fibrosis	Absent	337	77	124	136	22.8	36.8	40.4	*ns*
	Present	42	10	14	18	23.8	33.3	42.9	
	*n*	379							
Tumour border	Infiltrative	324	72	114	138	22.2	35.2	42.6	*ns*
	Pushing	55	15	24	16	27.3	43.6	29.1	
	*n*	379							
Ki67 expression (20% threshold)	Low	307	73	115	119	23.8	37.5	38.8	*ns*
	High	53	10	16	27	18.9	30.2	50.9	
	*n*	*360*							
HER2 status (CISH)	Negative	343	81	125	137	23.6	36.4	39.9	*ns*
	Positive	39	7	12	20	17.9	30.8	51.3	
	*n*	*382*							
ER status	Positive	285	58	110	117	20.4	38.6	41.1	**0.0035**
	Negative	82	28	17	37	34.1	20.7	45.1	
	*n*	*367*							
TN status	Non-TNBC	311	64	115	132	20.6	37.0	42.4	*ns*
	TNBC	68	22	22	24	32.4	32.4	35.3	
	*n*	*379*							
Other prognostic subgroups	HER2+	38	7	12	19	18.4	31.6	50.0	*ns*
	HR+/HER2-neg (Ki67-high)	235	56	88	91	23.8	37.4	38.7	
	HR+/HER2-neg (Ki67-low)	23	1	9	13	4.3	39.1	56.5	
	TN (basal-like)	54	18	17	19	33.3	31.5	35.2	
	TN (non-basal)	12	3	5	4	25.0	41.7	33.3	
	*n*	*362*							

*p* Values shown are from chi square or Fisher's exact tests.

**Table 2:** Prognostic value of nuclear SASH1 expression in ER-positive breast cancer over 25 years

Covariates	Univariate^a^			Multivariate^b^		
	*HR*	95% CI	*p* value	*HR*	95% CI	*p* value
HER2	3.23	1.29–8.07	< 0.0001	2.45	1.21–4.98	0.0134
Ki67	1.87	0.88–3.95	0.0325	2.38	1.88–4.76	0.0150
**SASH1**	**0.54**	**0.35–0.82**	**0.0068**	**0.45**	**0.27–0.77**	**0.0037**
Size	2.32	1.52–3.53	0.0002	1.97	1.97–3.22	0.0078
Grade	3.17	1.75–5.76	0.0085	-	-	*ns*

Univariate and multivariate analysis of factors associated with breast cancer-specific survival among 223 ER+ cases in the Queensland Follow-up Cohort.

(a) Kaplan-Meier analysis with the Log-rank test

(b) Stepwise Cox proportional hazards regression.

We also analysed relapse-free survival in ER+ cases with high and low relative expression of *SASH1* mRNA by meta-analysis of breast cancer gene expression data from the KM plotter database [18]. Consistent with our IHC analyses, higher expression of *SASH1* was associated with better outcome in ER+ cases of luminal A molecular subtype, but not luminal B (more proliferative) cases, and also in ER/PR+ but not ER+/PR- cases (Figure 6C). Multivariate analysis demonstrated that this association was independent of *ERBB2* (HER2) or *MKI67* (Ki67) expression (Table 3; *p* = 0.0032; HR 0.74).

**Table 3:** Multivariate Cox regression analysis of factors associated with 15 year breast cancer relapse-free survival in the KM Plotter database [18]

	ER-positive			HER2-positive			Triple-negative, basal-like		
Transcript	*HR*	95% CI	*p* value	*HR*	95% CI	*p* value	HR	95% CI	*p* value
*SASH1*	0.74	0.61–0.9	0.0032	3.07	1.71–5.51	0.0002	1.78	0.91–3.49	0.09
*MKI67*	1.39	1.09–1.75	0.0068			*ns*			*ns*
*ERBB2*	1.20	0.94–1.54	*ns*	-	-	-	-	-	-
*ESR1*	-	-	*-*	0.55	0.32–0.97	0.038	-	-	-

ER, PR and HER2 status was determined using clinical diagnostic criteria. Expression of *SASH1*, *MKI67* (Ki67), *ERBB2* (HER2) and *ESR1* (ER) mRNA was divided according to best statistical stratification. CI, confidence interval; HR, hazard ratio.

**Figure 7:**
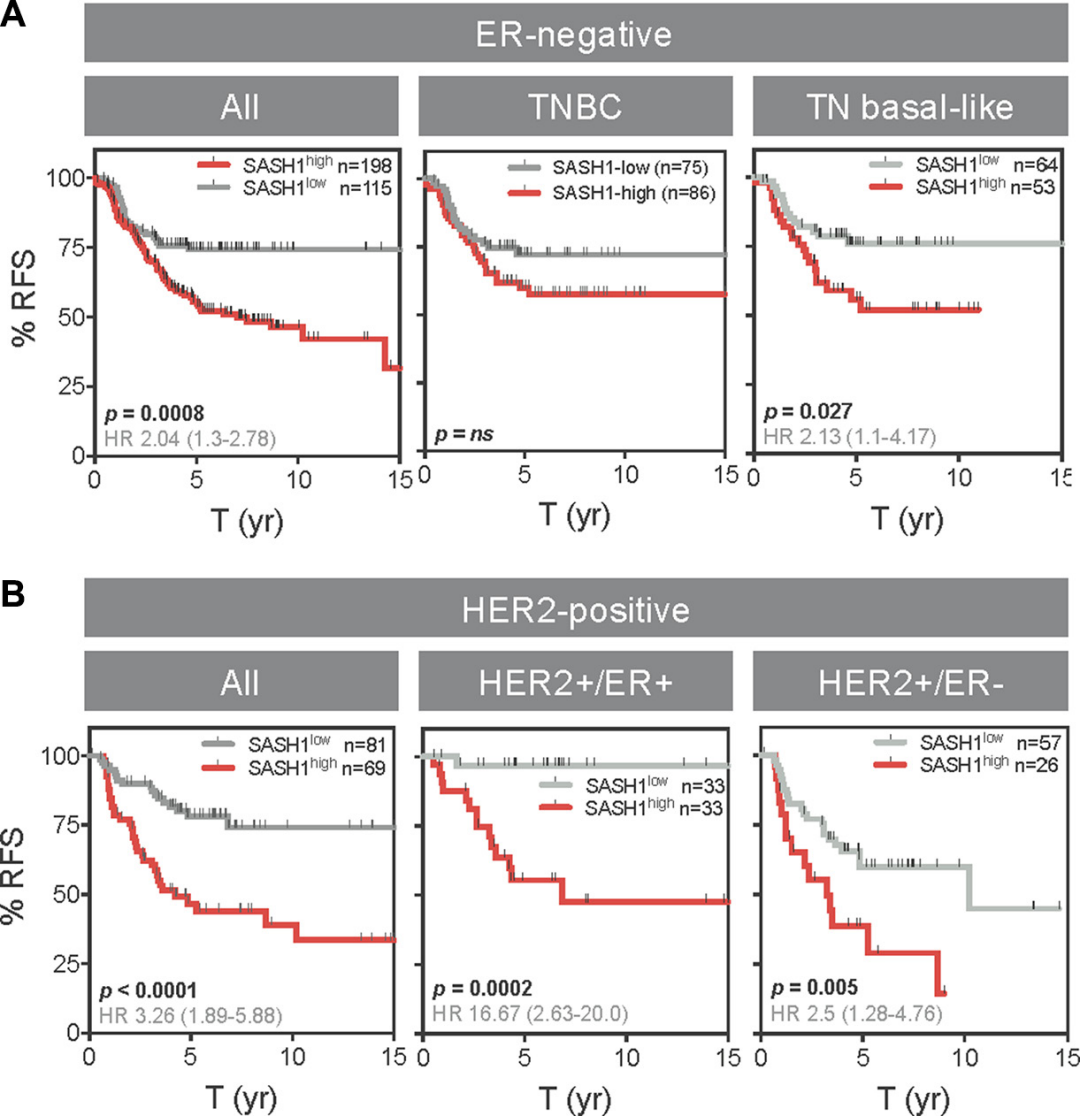
High SASH1 expression is an independent marker of poor prognosis in ER-negative and HER2-positive breast cancer subtypes Kaplan-Meier analysis of relapse-free survival (RFS) according to high or low *SASH1* mRNA expression (KM Plotter database). (**A**) ER-negative subgroups. (**B**) HER2+ subgroups. ER and HER2 status defined by clinical diagnostic criteria. Log-rank *p* values and hazard ratios (HR; 95% confidence intervals in parentheses) are indicated.

Interestingly, *SASH1* mRNA expression was inversely associated with relapse-free survival ER-negative and triple-negative basal-like cases (TNBL; Figure 7A), though this was not significant in a multivariate model (Table 3). Similarly, *SASH1* was inversely associated with outcome in HER2+ disease (Figure 7B; *p* < 0.0001; HR 3.26), independent of Estrogen Receptor 1 (ESR1) and *MKI67* (Table 3; *p* = 0.0002; HR 3.07). The relationship was particularly striking for HER2+/ER+ cases where low *SASH1* was associated with nearly 100% survival (*p* = 0.0002; HR 0.06). TNBL and HER2+ subgroup sizes in the QFU cohort were not conducive to statistical analysis (*n* = 54 and 38, respectively).

## DISCUSSION

Based on its association with favourable prognosis in multiple human malignancies [2–8, 19] and adverse effects on cancer cell line viability and invasiveness *in vitro* [3, 6–8], SASH1 has been proposed as a tumour suppressor [2–4, 6, 8, 14, 20, 21]. Consistent with this, we found that ectopic expression of SASH1 reduced breast cancer cell line viability, and so we hypothesised it could be involved in processes required for maintaining cancer cell viability *in vivo*, and that increasing its expression could be a novel treatment strategy.

To identify drug candidates with this capability, we performed *in silico* connectivity mapping using the cmap database (Broad Insitute [15]) and identified the antihistamine chloropyramine as a candidate SASH1 inducer. Others have shown that chloropyramine reduces survival of cell lines from melanoma, neuroblastoma, breast and pancreatic cancers, involving inhibition of FAK and VEGFR3 signalling [22–26]. Interestingly, SASH1 has also been implicated in modulation of FAK signalling [9]. Consistent with the connectivity screen, chloropyramine induced SASH1 expression in seven of the eight breast cancer cell lines tested, and reduced the viability of six lines. Transfecting the three most sensitive lines with *SASH1* siRNA prior to treatment partially rescued the cytotoxic response, suggesting that chloropyramine-induced cancer cell line death is at least partly mediated by SASH1. There is at least one existing report suggesting that chloropyramine can reduce breast cancer xenograft growth *in vivo* [26], though additional preclinical studies are required to more comprehensively characterise the anti-tumour activity of the agent.

If chloropyramine were to be considered for therapy, it would be important to establish which patient group may benefit. The prognostic significance of SASH1 expression is less characterised in breast cancer compared with other malignancies, so we investigated this using two clinically-annotated tumour cohorts. Overall we found that SASH1 was associated with favourable prognosis, but stratifying the cases based on ER status revealed that this was driven by the more prevalent ER-positive cases (75–80% of the cohorts analysed), and SASH1 expression was inversely associated with outcome in ER-negative, TNBL and HER2+ breast cancers. Thus although SASH1 has been coined a tumour suppressor, our findings suggest that this may be oversimplifying its role and that context is critical. Indeed, interrogating other KM Plotter cancer datasets we found strong associations between *SASH1* mRNA expression and better overall survival in lung cancer but very poor outcome in gastric cancer (data not shown).

Breast cancer management has improved substantially over the last few decades, but in Australia, the US and UK, 15–20% of patients still do not survive 10 years after diagnosis [27, 28]. This amounts to a large proportion of cancer-related morbidity and mortality and cost to the public health sector. Tumours that do not respond to current first-line therapies are likely to be more complex and heterogeneous. Disease control in the future will depend on an increased understanding of the molecular biology of the disease, leading to identification of novel personalised medicine therapy approaches linked to companion diagnostics. *In silico* connectivity screening provides a means to fast-track the identification of gene-drug associations and drug repurposing opportunities. *In silico* mapping of associations between induction of SASH1 and ‘off-the-shelf’ drugs identified a novel candidate, chloropyramine, with antitumour activity *in vitro*. Chloropyramine and other first-generation H1 antagonists are sedating because they cross the blood-brain-barrier, and were therefore superseded by peripherally-acting agents for the treatment of allergy. Given that brain uptake can be desirable in molecular oncology and chloropyramine is otherwise well-tolerated, the potential application for this or structurally related agents for low toxicity treatment of breast and other cancers deserves further mechanistic and preclinical investigation. Furthermore, phase 0 and dose-finding phase I, neoadjuvant, biomarker-driven clinical trials could allow us to confirm pharmacodynamic induction of SASH1 by chloropyramine that would underpin further repurposing studies of the agent in the future.

## MATERIALS AND METHODS

### Cell culture and transfection

Breast cancer cell lines were cultured at 37°C with 5% CO_2_ in RPMI with 10% FCS (MDA-MB-231, MDA-MB-361, T47-D, and BT-549), DMEM with 10% FCS (MCF7, MDA-MB-468, and Hs578T) or Ham's F12 with 5% FBS and 10 μg/ml recombinant human epidermal growth factor (SUM1315). Insulin was supplemented at 0.01 mg/mL for the MCF7, Hs578T, BT-549, T47-D, and SUM1315 cell lines. Cells were routinely passaged with trypsin and maintained at low passage. Chloropyramine (Sigma-Aldrich) was added to adherent cultured cells 24 hours after seeding at the indicated concentrations (0–100 μM). Cell line authentication by STR profiling was performed at QIMR Berghofer, with comparison to Children's oncology group cell culture and Xenograft repository (http://www.cogcell.org).

For siRNA experiments, esiRNAs (Sigma) targeting SASH1 or non-specific control oligos were transfected using RNAiMax (Invitrogen) as per the manufacturer's instructions. Double-transfections were performed 24 hours apart and samples were analysed 72 hours after the initial transfection where optimal SASH1 depletion was observed. For overexpression studies, the full-length *SASH1* cDNA was cloned into the mammalian expression vector pCMV6 (Origene). Three μg of DNA (SASH1-GFP or GFP) and 6 μL of Lipofectamine 2000 (Invitrogen) were used to transfect cells in a T25 flask, as per the manufacturer's instructions. Cells were harvested 24–48 h post-transfection for optimal overexpression and death assessment as indicated in figure legends.

### Immunoblotting

Immunoblotting was carried out as described previously [29]. Briefly, cells were lysed (20 mM Hepes pH 8.0, 150 mM KCl, 5% glycerol, 10 mM MgCl_2_, 0.5 mM EDTA, 0.02% NP-40, freshly supplemented with NaF, NaVO_4_, PMSF and protease inhibitors) and sonicated. Lysates were cleared by centrifugation and protein concentrations were estimated using the Bradford assay (Bio-Rad). Typically 50 μg of protein lysate was resolved on Bolt 4–12% gradient gels (Invitrogen) and proteins were transferred to nitrocellulose membrane (Bio-Rad). Membranes were blocked in 2% fish skin gelatin, 1% tween-20 in PBS (Sigma) for 1 h and incubated with primary antibodies overnight 4°C in the same buffer. Following incubation with secondary antibodies, membranes were visualised using a Li-COR Odyssey infrared scanner. Fluorescence intensity was quantified relative to a loading control (β-actin or Histone H3) using Image J software.

### Cell death assay

Following incubation of cells with the indicated treatments, propidium iodide (10 μg/ml) and Hoechst (1 μg/ml) were added 30 min before imaging. Cells were imaged on an IN Cell Analyzer 2200 (GE Healthcare; 10× objective). Live/dead cell analysis was performed using IN Cell analysis software.

### Cell confluency assay

Cells were seeded at 2,500 cells per well in 96 well plates (Nunc). Cells were allowed to adhere for 24 h before chloropyramine addition and then imaged every 2 hours for 96 hours in an IncuCyte ZOOM^®^ live cell imager (Essen Bioscience) to calculate confluence.

### Annexin V/Propidium iodide (PI) analysis

Annexin V/PI staining was carried out as described previously [30]. Briefly, treated or untreated cells (adherent and floating) were harvested using trypsin and centrifugation, washed in PBS and then stained according to the Promega Annexin V-FITC apoptosis detection kit protocol. Annexin V-positive (apoptotic) cells were detected using a Gallios flow cytometer system and quantified with Flow Jo software.

### Drug screen with connectivity mapping

A gene expression connectivity mapping approach was employed to identify candidate compounds that may induce SASH1 expression. SASH1 was mapped to Affymetrix HG-U133A probeset IDs to form a query gene signature. This was compared to the reference drug expression profiles in the CMap02 database using the sscMap algorithm [31, 32]. Compounds with statistically significant positive connection to the query gene signature were selected as candidate SASH1 inducing drugs for further laboratory validation as described.

### Immunohistochemistry (IHC) and tissue microarray (TMA) analysis

SASH1 protein expression in breast cancer was investigated by IHC analysis of the Queensland follow-up (QFU) resource, comprising TMAs of 449 invasive breast carcinomas (sampled in duplicate) and associated clinical data, including survival outcomes of over 20 years [33]. The use of patient data and clinical samples in this study were approved by human research ethics committees of the University of Queensland and the Royal Brisbane and Women's Hospital (RBWH).

Four μm TMA sections were processed in a decloaker for antigen retrieval in EDTA buffer (pH 8.8) for 15 minutes, and then IHC was performed using an anti-SASH1 antibody (Sigma Prestige HPA029947; 1:850), and the Mach 1 Universal HRP-Polymer Detection kit (Biocare Medical). Haematoxylin-counterstained, mounted sections were then scanned at 40 x magnification on an Aperio AT Turbo slide scanner (Leica Biosystems). Digital images of individual tissue cores were scored by a qualified Pathologist (AMM) according to tumour cell nuclear staining intensity. Using the maximum score of duplicate tissue cores for each case, associations between SASH1 expression and clinicopathologic variables were investigated using chi-square and log-rank tests (GraphPad Prism v6).

### *In silico* analysis of *SASH1* mRNA prognostic significance

The relationships between *SASH1* mRNA expression and relapse-free survival were analysed using the KM plotter breast cancer database [18]. Three different *SASH1* probes were analysed; representative data from the ‘JetSet’ optimal probe are presented in this paper [34].

### Statistical analyses

Most statistical tests were performed using Graph Pad Prism V6. Associations between breast tumour SASH1 expression and clinicopathologic variables were investigated using chi square or Fisher's exact tests. Relationships between breast tumour SASH1 expression and relapse-free or overall survival were represented with Kaplan-Meier curves and analysed using the log-rank test. Analysis of SASH1-mediated changes in apoptosis and proliferation, and SASH1 expression following chloropyramine treatment were investigated with students two-tailed *t*-tests. For multivariate analysis of *SASH1* mRNA prognostic significance was performed using the parameters available (*MKI67* and *ERBB2* expression) and inbuilt function in the KM plotter database [18].

For multivariate analysis of SASH1 protein prognostic significance in ER+ breast cancer, we performed stepwise Cox regression analysis using MedCalc^®^ software (v13.2) including HER2 status (determined by SISH according to diagnostic criteria), Ki67 status (nuclear staining in at least 20% tumour cells), histological grade (assessed by an experienced Pathologist (SRL) and tumour size (derived from clinical pathology reports). These data were complete for 223 ER+ cases. *p* Values > 0.05 were considered significant.
